# Immune checkpoint inhibitor-induced refractory polyarthritis rapidly improved by sarilumab and monitoring with joint ultrasonography: A case report

**DOI:** 10.1097/MD.0000000000028428

**Published:** 2022-01-14

**Authors:** Kazuya Abe, Yuichi Ishikawa, Michio Fujiwara, Hiroko Yukawa, Takeshi Yanagihara, Saori Takei, Hitoshi Arioka, Yasuhiko Kita

**Affiliations:** a Department of Rheumatology, Yokohama Rosai Hospital, 3211 Kozukue-cho, Kohoku-ku, Yokohama, Kanagawa, Japan; b Department of Allergy and Clinical Immunology, Chiba University Hospital, 1-8-1 Inohana, Chou-ku, Chiba, Japan; c Sato Clinic, 4-28-5 Ebisu, Shibuya-ku, Tokyo, Japan; d Department of Medical Oncology, Yokohama Rosai Hospital, 3211 Kozukue-cho, Kohoku-ku, Yokohama, Kanagawa, Japan.

**Keywords:** arthritis, immune checkpoint inhibitor, immune-related adverse event, interleukin-6, sarilumab

## Abstract

**Rationale:**

Immune checkpoint inhibitors (ICIs) have shown efficacy for the treatment of various kinds of malignant tumors. However, ICIs can cause immune-related adverse events, such as arthritis. Nevertheless, the treatment of ICI-induced arthritis has not been established yet. Here we report a case of ICI-induced polyarthritis successfully treated using sarilumab and monitored using joint ultrasonography.

**Patient concerns:**

A 61-year-old man presented with polyarthritis. He had been treated with nivolumab for recurrent renal cell carcinoma 11 months before. He developed ICI-induced nephritis (proteinuria and elevated serum creatinine) 3 months before, which resolved after discontinuing nivolumab for 1 month. Two months after resuming nivolumab, he developed polyarthralgia and joint swelling, which were suspected to be associated with nivolumab administration, and hence we discontinued nivolumab again. Laboratory tests revealed elevated C-reactive protein level and erythrocyte sedimentation rate, but were negative for rheumatoid factor and anti-cyclic citrullinated peptide antibody. Joint ultrasonography revealed active synovitis in several joints, but a joint X-ray revealed no bone erosion.

**Diagnoses:**

We diagnosed polyarthritis as ICI-induced arthritis because the findings were not typical of rheumatoid arthritis (no bone erosion and seronegativity) and the patient had already developed other immune-related adverse events (ICI-induced nephritis).

**Interventions:**

After discontinuation of nivolumab, we started treatment with 15 mg daily prednisolone and 1000 mg daily sulfasalazine, although it was ineffective. Hence, we initiated 200 mg biweekly sarilumab.

**Outcomes:**

Following sarilumab administration, polyarthritis improved rapidly, and joint ultrasonography confirmed the rapid improvement of synovitis. Hence, we tapered off the glucocorticoid treatment. No recurrence of renal cell carcinoma was noted for 2 years after the initiation of sarilumab despite no anti-tumor therapy.

**Lessons:**

Sarilumab may serve as a good treatment option for treating refractory ICI-induced polyarthritis. Joint ultrasonography may contribute to the evaluation of ICI-induced polyarthritis and monitoring the effects of treatments.

## 1 Introduction

Recently, immune checkpoint inhibitors (ICIs) have been frequently used for treating various malignant tumors. Despite the efficacy of ICIs for treating various types of tumors, they can cause immune-related adverse events (irAEs).^[1]^ IrAEs are induced by the excessive enhancement of the immune system by ICIs and can result in inflammatory organ damage. Although the pathogenesis of irAEs has not been completely elucidated, it is considered to be associated with T-cell activation and increased inflammatory cytokine production.^[2]^ IrAEs are reported to cause various symptoms, including rheumatological complications. The incidence of ICI-induced inflammatory arthritis, which resembles rheumatoid arthritis (RA), is reportedly 1% to 43%.^[3]^

However, the diagnostic and evaluation criteria for ICI-induced arthritis have not been established yet. Moreover, the treatment strategies for ICI-induced arthritis remain to be established completely and are still controversial.^[4]^ A few case series have suggested that tumor necrosis factor inhibitor (TNFi) is effective for treating ICI-induced arthritis,^[5,6]^ and an interleukin (IL)-6 receptor antagonist is also reported to be effective.^[7]^

Herein, we report the case of a patient who developed polyarthritis after nivolumab treatment. We also report on the diagnostic efficacy of joint ultrasonography and disease activity evaluation. Polyarthritis was refractory to treatment with glucocorticoid and sulfasalazine. Therefore, we initiated the administration of sarilumab, an interleukin-6 (IL-6) receptor antagonist, which resulted in the rapid improvement of polyarthritis. Written informed consent was obtained from the patient for the publication of this case report.

## 2 Case report

The patient was a 61-year-old man presenting with polyarthralgia. He had no rheumatic and autoimmune diseases, but he had been diagnosed with renal cell carcinoma (clear cell carcinoma, pT3bN0M0, stage III) and had undergone right radical nephrectomy 9 years ago. One year after surgery, lung and mediastinal lymph node metastases were observed. Thus, molecular targeted therapy (sunitinib, a vascular endothelial growth factor tyrosine kinase inhibitor, 50 mg orally once daily for 4 weeks on, and 2 weeks off in each 6-week cycle) was started.

Seven years after the sunitinib, metastasis to the pancreas, adrenal gland, and bone appeared. A distal pancreatectomy was performed on him, and we believed switching to a drug with a different mechanism of action would be appropriate. Therefore, we replaced sunitinib with nivolumab (240 mg biweekly), which achieved a partial response. However, 3 months after nivolumab administration, the serum creatinine level increased from 0.90 to 1.67 mg/dL, and proteinuria was >1.0 g/d.

**Figure 1:**
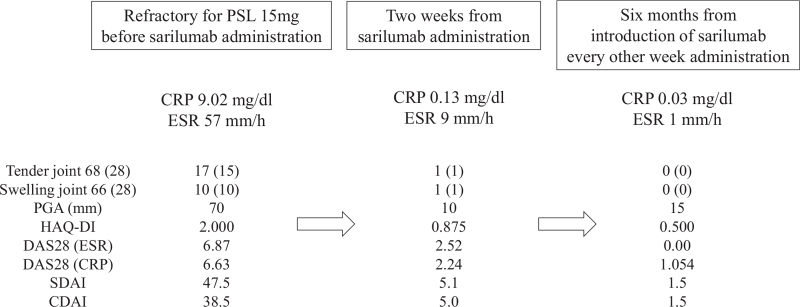
Clinical course after sarilumab administration. Tenderness and swelling were assessed in 68 and 66 joints, respectively. Tender and swollen joints (approximately 28 joints) were assessed according to the following criteria: DAS28-CRP/ESR, CDAI, and SDAI. Glucocorticoids were tapered off 3 months after sarilumab administration. CDAI = clinical disease activity index,^[11]^ CRP = C-reactive protein, DAS = disease activity index,^[12,13]^ ESR = erythrocyte sedimentation rate, HAQ-DI = Health Assessment Questionnaire Disability Index,^[14]^ PGA = patient's global assessment, SDAI = simplified disease activity index.^[11]^

**Figure 2:**
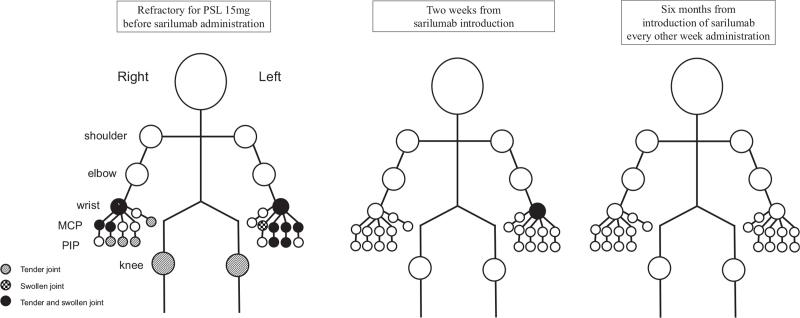
Clinical course about affected joints after sarilumab administration. MCP = metacarpophalangeal joints, PIP = proximal interphalangeal joints.

ICIs have been reported to induce nephritis, which leads to renal impairment and proteinuria. The clinical features of ICI-induced nephritis are more frequent in men, and the time of onset is ∼3 months.^[8,9]^ We suspected that the kidney injury in our case was ICI-induced nephritis. Therefore, we considered performing renal biopsy. However, because the right kidney had previously been removed, we decided against biopsy considering that solitary kidney is a contraindication for renal biopsy.^[10]^ Thus, nivolumab was discontinued, 1 month after which ICI-induced nephritis resolved. Hence, we subsequently resumed treatment with the same dose of nivolumab, but 2 months later (7 months from initial nivolumab administration), the patient developed polyarthralgia and had elevated C-reactive protein levels (Figs. 1 and 2). Laboratory tests were negative for rheumatoid factor and anti-cyclic citrullinated peptide antibody (Table 1).

**Table 1:** Laboratory examination when arthritis developed.

<Complete blood count>			<Biochemistry>			<Serological>		
White blood cell	10,500	/μL	T-Bil	0.26	mg/dL	RF	4	IU/mL
Segmented	74	%	ALP	265	U/L	ACPA	<0.5	U/mL
Lymphocyte	10	%	γ-GTP	85	U/L	ANA	<40	×
Monocyte	5	%	AST	15	U/L	Anti-SS-A	<1.0	U/mL
Eosinophils	9	%	ALT	11	U/L	Anti-SS-B	<1.0	U/mL
Hemoglobin	10.9	g/dL	LDH	191	U/L	MPO-ANCA	<1.0	U/mL
Platelets	38.3 × 10^4^	/μL	CPK	63	U/mL	PR3-ANCA	<1.0	U/mL
<Urinalysis >			Tp	6.8	g/dL	Erythrocyte sedimentation rate	57	mm/h
pH	5.5		Alb	2.9	g/dL			
Protein	(1+)		BUN	18	mg/dL			
Red blood cells	(–)		Cr	0.96	mg/dL			
White blood cells	(–)		Na	138	mEq/L			
Pathologic cells	(–)		K	4.3	mEq/L			
			Cl	99	mEq/L			
			CRP	9.02	mg/dL			

γ-GTP = γ-glutamyltransferase, ACPA = anti-citrullinated peptides antibody, Alb = albumin, ALP = alkaline phosphatase, ALT = alanine aminotransferase, ANA = antinuclear antibody, Anti-SS-A = Anti SS-A antibody, Anti-SS-B = Anti SS-B antibody, AST = aspartic aminotransferase, BUN = blood urea nitrogen, CPK = creatine phosphokinase, Cr = creatinine, CRP = C-reactive protein, LDH = lactate dehydrogenase, MPO-ANCA = myeloperoxidase anti-neutrophil cytoplasmic autoantibodies, PR3-ANCA = proteinase 3 anti-neutrophil cytoplasmic autoantibodies, RF = rheumatoid factor, T-Bil = total bilirubin, TP = total protein.

Joint ultrasonography showed active synovitis in several joints. Although the patient seemed to fulfill the criteria for RA,^[15]^ we finally diagnosed ICI-induced polyarthritis owing to negative findings for rheumatoid factor and anti-cyclic citrullinated peptide antibody, absence of bone erosion on joint X-ray, and the irAE (nephritis), which had already developed after ICI administration. We discontinued nivolumab again, and because a nonsteroidal anti-inflammatory drug (loxoprofen) did not improve his polyarthritis, we started treatment with 15 mg daily prednisolone (PSL). However, despite 2 weeks of PSL administration, his polyarthritis worsened. Therefore, we added 1000 mg daily sulfasalazine to this regimen, which was also ineffective in controlling his polyarthritis. Increasing the glucocorticoid dose was considered. However, we avoided administering high doses of glucocorticoids because he was assumed to be an immunocompromised patient by nephrectomy and chemotherapy. We therefore reasoned that he would be adversely affected more by the strong side effects of glucocorticoids, such as infection.

**Figure 3:**
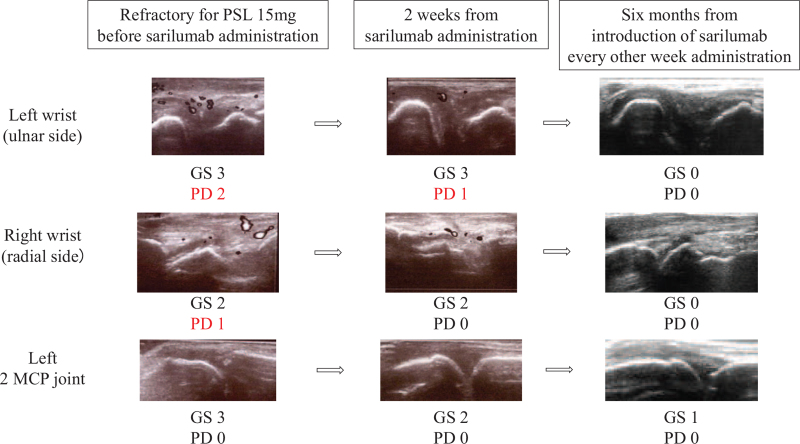
Course of the amelioration of immune checkpoint inhibitor-induced arthritis assessed using joint ultrasonography. GS = gray-scale grade, MCP = metacarpophalangeal joints, PD = power Doppler grade.^[16]^

Therefore, we decided to initiate treatment with 200 mg biweekly sarilumab. After 2 weeks from sarilumab, acute phase reactants (C-reactive protein and erythrocyte sedimentation rate) were decreased and disease activity measures were also improved, which meant rapid improvement polyarthritis (Fig. 1). Strategies for monitoring ICI-induced arthritis are not yet established. We therefore used joint ultrasonography as an assessment tool, which confirmed the rapid improvement of synovitis (Fig. 3). Sarilumab administration was continued, and glucocorticoid was tapered and discontinued 4 months after sarilumab administration. After discontinuing the glucocorticoid, remission of arthritis was maintained. Further, there was no recurrence of renal cell carcinoma, for 2 years after the starting treatment with sarilumab despite no anti-tumor therapy.

## 3 Discussion

Joint ultrasonography is a helpful assessment and monitoring tool for ICI-induced polyarthritis. In addition, sarilumab might be a good therapeutic option and show rapid effectiveness against refractory ICI-induced arthritis. ICI-induced arthritis cannot be considered as a rare irAE considering that its incidence is 46%.^[3]^ ICI-induced arthritis was previously reported to develop after 6.3 ± 4.3 months from first ICI administration.^[17]^ In our case, polyarthritis occurred 7 months after nivolumab administration, which corroborates the findings of the previously mentioned report.

ICI-induced polyarthritis is sometimes refractory, and the treatment of refractory and severe ICI-induced polyarthritis remains controversial. The recommended treatment strategy for ICI-induced arthritis involves treatment with 0.5 to 1.0 mg/kg/d PSL. If glucocorticoid monotherapy does not improve arthritis, TNFi is recommended.^[4]^ The problem with this strategy is that a long period of high-dose glucocorticoid administration is required, which may cause AEs, such as osteoporosis, diabetes, or infection.^[18]^ Furthermore, TNFi may suppress the immune response to malignant tumors.^[19]^ Therefore, ICI-induced activation of tumor immunity may also be suppressed by TNFi. The IL-6 receptor antagonist tocilizumab is reported to be effectiveness against ICI-induced arthritis.^[7]^ Further, IL-6 has been shown to promote cancer occurrence, progression, and metastasis, but IL-6 inhibition impedes cancer progression.^[20,21]^ The pathophysiology of irAEs of IL-6 receptor antagonists is thought to be mediated by the suppression of T helper 17 cells. IL-6 inhibition does not inhibit CD8-positive T cells, which has an antitumor effect; thus, IL-6 is thought to have less effect than ICIs on malignant tumors.^[22]^ Accordingly, IL-6 receptor antagonists might be effective against irAEs.

In this case, we selected sarilumab as an IL-6 receptor antagonist because its efficacy for RA is well-established and sarilumab administration can be initiated using its maximum dose to achieve rapid improvement.^[23]^ Therefore, we thought that sarilumab could relieve refractory ICI-induced arthritis rapidly, similar to RA. We found sarilumab administration to be effective as expected and could promptly taper glucocorticoids.

We performed joint ultrasonography to diagnose and evaluate arthritis. It has been demonstrated that joint ultrasonography helps in the diagnosis and evaluation of RA.^[24,25]^ It has also been reported that ICI-induced synovitis can be diagnosed via joint ultrasonography in a similar manner to RA.^[26]^ Patients with cancer sometimes have musculoskeletal pain induced by bone metastasis or tumor-induced pain. Therefore, joint ultrasonography might help to accurately diagnose ICI-induced synovitis. We followed up synovitis using joint ultrasonography and could thus quickly monitor any improvement in arthritis. As joint ultrasonography can rapidly recognize improvement in arthritis, it might also help to avoid excessive immunosuppressive therapy for ICI-induced arthritis.

## 4 Conclusions

Sarilumab may serve as an effective treatment option for treating refractory ICI-induced polyarthritis. Joint ultrasonography may contribute to the evaluation of ICI-induced polyarthritis and monitoring the effects of treatments.

## Acknowledgments

The authors gratefully acknowledge the work of past and present members of our hospitals.

## Author contributions

**Conceptualization:** Kazuya Abe, Yuichi Ishikawa, Hiroko Yukawa.

**Investigation:** Kazuya Abe.

**Writing – original draft:** Kazuya Abe.

**Writing – review & editing:** Yuichi Ishikawa, Michio Fujiwara, Hiroko Yukawa, Takeshi Yanagihara, Saori Takei, Hitoshi Arioka, Yasuhiko Kita.
